# A descriptive study of acute outbreaks of respiratory disease in Norwegian fattening pig herds

**DOI:** 10.1186/s13028-020-00529-z

**Published:** 2020-06-24

**Authors:** Liza Miriam Cohen, Carl Andreas Grøntvedt, Thea B. Klem, Stine Margrethe Gulliksen, Birgit Ranheim, Jens Peter Nielsen, Mette Valheim, Camilla Kielland

**Affiliations:** 1 Department of Production Animal Clinical Sciences, Faculty of Veterinary Medicine and Biosciences, Norwegian University of Life Sciences (NMBU), P.O. Box 8146 Dep, 0033 Oslo, Norway; 2 Norwegian Veterinary Institute, P.O. Box 750 Sentrum, 0106 Oslo, Norway; 3 Animalia, P.O. Box 396, Økern, 0513 Oslo, Norway; 4 Department of Veterinary and Animal Sciences, Faculty of Health and Medical Sciences, University of Copenhagen, Grønnegårdsvej 2, 1870 Frederiksberg C, Denmark

**Keywords:** *Actinobacillus pleuropneumoniae*, Acute respiratory disease, Fattening pigs, Outbreak diagnostics

## Abstract

**Background:**

Respiratory diseases are major health concerns in the pig production sector worldwide, contributing adversely to morbidity and mortality. Over the past years there was a rise in reported incidents of respiratory disease in pigs in Norway, despite population wide freedom from Aujeszky´s disease, porcine reproductive and respiratory syndrome, porcine respiratory corona virus and enzootic pneumonia. The main objective of this study was to investigate acute outbreaks of respiratory disease in conventional Norwegian fattening pig herds. The study included 14 herds. In seven herds with reported outbreaks of acute respiratory disease, data on clinical signs was recorded and samples for laboratory examination were collected. Diagnostic protocols were compared by parallel analysis of clinically healthy pigs from seven non-outbreak herds.

**Results:**

The most commonly reported clinical signs were sudden deaths and dyspnea. An average compartment morbidity of 60%, mortality of 4% and case fatality of 9% was recorded in the outbreak herds. Post-mortem examinations revealed acute lesions resembling porcine pleuropneumonia in all 28 pigs investigated from the outbreak herds and in 2 of the 24 (8%) pigs from the non-outbreak herds. Chronic lesions were recorded in another 2 pigs (8%) from the non-outbreak herds. *Actinobacillus pleuropneumoniae* serovar 8 was isolated from lungs and/or pleura from all tested pigs (n = 28) in the outbreak herds, and from 2 out of 24 pigs (8%) in the non-outbreak herds, one pig with an acute and another pig with a chronic infection. No other significant bacterial findings were made. Seroconversion to *A. pleuropneumoniae* antibodies was detectable in all outbreak herds analyzed and in six out of seven non-outbreak herds, but the risk ratio for seroconversion of individual pigs was higher (risk ratio 2.3 [1.50- 3.43 95% CI; P < 0.001]) in the outbreak herds. All herds tested positive for porcine circovirus type 2 and negative for influenza A viruses on oral fluid RT-qPCR.

**Conclusion:**

The main etiological pathogen found during acute outbreaks of respiratory disease was *A. pleuropneumoniae* serovar 8. All pigs from outbreak herds had typical lesions of acute porcine pleuropneumonia, and only *A. pleuropneumoniae* serovar 8 was identified. Co-infections were not found to impact disease development.

## Background

Respiratory diseases give rise to major health concerns in pig populations worldwide. They are believed to contribute adversely to morbidity and mortality, increased use of antimicrobials, poor pig welfare and reduced productivity [1–3]. The direct effect of disease on these parameters are however hard to determine in field conditions. Studies show that coinfections with different respiratory agents are common in pigs [4, 5]. Viral infections often predispose for secondary bacterial infections. This has been studied under experimental conditions, i.e. coinfections of porcine reproductive and respiratory syndrome virus (PRRSV) and *Mycoplasma hyopneumoniae* [6], PRRSV and *Actinobacillus pleuropneumoniae* [7], swine influenza virus (SIV) and *Bordetella bronchiseptica* [8]. Moderate to marked fever, lethargy, coughing, sneezing and dyspnea are common clinical signs during disease outbreaks [9, 10]. The presence of multiple pathogens often increases the severity of disease and occurrence of lesions in the respiratory tract [8, 11, 12]. There are differences in occurrence and distribution of pathogens between countries, regions and herds [13, 14] that contribute to the complexity of respiratory disease.

Due to strict import regulations in Norway, there is negligible import of live pigs to the commercial pig population [15]. The national yearly yield was approximately 1.6 million slaughtered pigs in 2018, originating mainly from 2580 registered fattener pig herds with a concession limit of maximum 2100 slaughtered pigs per year [16, 17]. The Norwegian pig production is also characterized by stringent regulation of antimicrobial drug use and a tradition of eradicating diseases from animal populations [18, 19]. The commercial pig population in Norway has documented freedom from several important respiratory pathogens including Aujeszky’s disease virus, PRRSV, SIV (apart from influenza A [H1N1]pdm09) [20] and *M. hyopneumoniae* [18]. After the pandemic in 2009/2010, antibodies to SIV (H1N1)pdm09 have been detected regularly from 25 to 50% of examined herds in Norway [21], but SIV (H1N1)pdm09 infections in the Norwegian pig population has been considered to have limited clinical impact [22]. In cases of respiratory disease in Norwegian herds, *A. pleuropneumoniae* has regularly been isolated from lungs of carcasses submitted for routine diagnostics [23]. Several studies from other countries conclude that *A. pleuropneumoniae* is normally present in most conventional pig herds, having a main reservoir in the tonsils of carrier pigs [24, 25]. Accordingly, outbreaks in conventional herds are most often triggered by factors related to animal housing, management and environment rather than an introduction of the bacteria in a naïve herd [26]. Preceding infection with a primary viral pathogen is also a possible triggering factor [4]. In the years between 2010 and 2014 there was an increase in reported acute cases of respiratory disease requiring veterinary treatment in Norway [27]. A systematic investigation of porcine respiratory disease outbreaks in Norway has not recently been performed, and updated knowledge is needed for appropriate disease prevention and intervention. The main objective of this study was to investigate clinical outbreaks of acute respiratory disease in Norwegian fattening pig herds, using a group of non-outbreak herds to compare diagnostic procedures.

## Methods

### Study design

#### Source population

The source population was the conventional fattening pig herds located in central and southern parts of Norway in the period between September 2017 and October 2018. The conventional herds are not part of the Norwegian Specific Pathogen Free (SPF) sub-population, in which herds are free from e.g. toxin producing *Pasteurella* *multocida* and all serotypes of *A.* *pleuropneumoniae*.

#### Sample population

Seven conventional fattening pig herds with acute outbreaks of respiratory disease (outbreak herds) and seven pig herds without respiratory disease outbreaks (non-outbreak herds) were included in this study.

The inclusion criteria for outbreak herds were; three or more pigs displaying acute signs of respiratory disease including fever and coughing and/or dyspnea, and/or otherwise reduced general condition e.g. lethargy or inappetence. Non-outbreak herds inclusion criteria were; absence of acute clinical signs of respiratory disease at the time of sampling, situated in the same geographical area as the outbreak herds. The non-outbreak herds were not matched to the outbreak herds by means of other parameters. Herds were included only if there were more than three weeks until planned slaughter, due to follow-up sampling per protocol. Two herds were excluded, due to treatment with antimicrobial drugs before sampling could be carried out, and insufficient time from outbreak to planned slaughter, respectively. Descriptive herd data are listed in Table 1.

**Table 1:** Overview of descriptive data in both outbreak and non-outbreak herds (n = 14)

Descriptive herd data	Outbreak herds (n = 7)^a^		Non-outbreak herds (n = 7)^b^	
	Median	Interquartile range	Median	Interquartile range
Production site	Single site production			
No. of suppliers	2	7	1	0
Herd size^c^	650	310	500	350
Yearly yield^d^	2109	1818	1543	1661
***Estimations from on-farm registrations:***				
Pigs in compartment^e^	155	90	196	255
Compartment volume per pig^f^	3.9 m^3^	2 m^3^	4.3 m^3^	1.5 m^3^
Floor space per pig^g^	1.0 m^2^	0.2 m^2^	1.1 m^2^	0.2 m^2^

^a^Herd type: 5 finishers, 2 farrow-to-finish. 6 herds: one compartment affected and tested. 1 herd: two compartments affected and tested, compartment average presented

^b^Herd type: 6 finishers, 1 farrow-to-finish. One compartment tested per herd

^c^Number of pigs in the herd/at the production site at the time of the outbreak/sampling

^d^Fattening pigs slaughtered over the last 12 months. Not considering piglets for sale

^e^Number of pigs the compartment with the ongoing outbreak

^f^m^3^ in the compartment divided by the number of pigs

^g^total m^2^ in the compartment divided by the number of pigs, not considering empty stalls, walkways etc

#### Recruitment and selection of herds

A network of veterinary practitioners was established to collect samples and herd data. The practitioners were contacted through emails, letters, meetings and announcements in relevant journals and national newspapers. The veterinarians contacted the project group immediately upon being called out to examine pigs with symptoms of acute respiratory disease. Outbreak herds were recruited for participation by the veterinary practitioners after meeting the inclusion criteria. Non-outbreak herds were then recruited by the veterinary practitioners contacting herd owners meeting the matching criteria, asking their participation and arranging a visit. Complete kits containing materials and detailed instructions for sample collection, preservation and transport were pre-distributed to designated pick up points at abattoirs and veterinary practice offices and sent to veterinarians across the country upon request.

### Herd visits

**Fig. 1:**
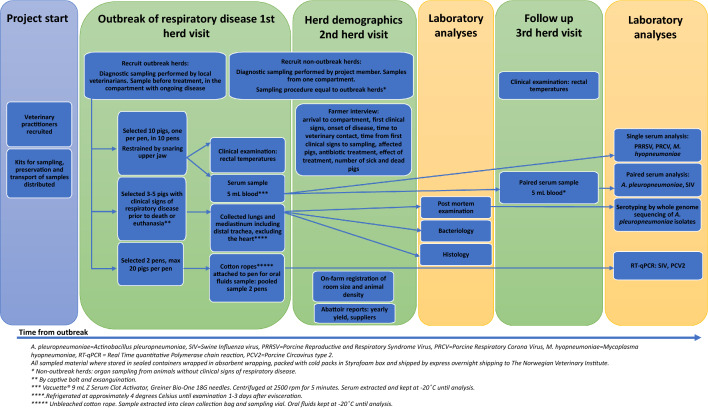
Overview of the timeline and procedure of the study

Each outbreak herd was visited on three occasions (Fig. 1, green boxes); the first visit was conducted as soon as possible during the reported outbreak for initial sampling. The second visit was performed 2 to 5 days later to conduct interviews and register herd demographic data. During the third and final visit two to four weeks after the first, follow-up samples were collected, as described in Fig. 1. Non-outbreak herds were visited on two occasions, once for initial sampling, farmer interviews and herd registrations, and secondly for follow up sampling.

#### First visit (outbreak sampling)

Details about the diagnostic sampling are shown in Fig. 1. Diagnostic sampling in outbreak herds was performed the day the veterinarian was notified about the disease. The veterinary practitioner reported observed clinical signs on a standardized submission form. In these herds, three to five pigs were selected for organ collection, 28 pigs were sampled in total. The selection was made from pigs with clinical signs of respiratory disease prior to death or euthanasia by captive bolt and exsanguination. Short time from death to sampling was considered, no additional criteria for sampling were applied. In non-outbreak herds three to five pigs were haphazardly selected, 24 pigs were sampled in total. Lungs and mediastinum (including pericardium, excluding the heart) and trachea caudal to the thoracic inlet were collected. Within each herd, care was given not to sample pigs treated with any antimicrobial drugs up to 14 days prior to the sampling.

Blood sampling was performed on a total of 10 pigs per herd by haphazard selection from as many pens in the compartment as possible, up to 10 pens. A total of 141 pigs were sampled. The pigs were selected regardless of clinical presentation and restrained by snaring the upper jaw. During restraint the pigs were ear-tagged for individual identification at follow-up sampling during the final visit. Rectal temperature was measured, and blood samples were collected (details in Fig. 1).

Pooled oral fluid (OF) samples were collected from two haphazardly selected pens (n = 14 pooled OF samples from 28 pens) using chewing rope as described by Prickett et al.  [28]. Care was given to keep the stress of the animals during sampling to a minimum.

#### Second visit (interviews and on-farm demographic data sampling)

Demographic data sampling was obtained by interviewing the farmers using a purpose-built questionnaire, see details in Fig. 1. Relevant information regarding the disease outbreaks including information about the first 5 days after noticing the first clinical signs was registered in outbreak herds. The following data was registered: dates of the pigs’ arrival to compartment, a description of earliest observed clinical signs, onset of disease, time to veterinary contact, time from the first clinical signs to the initial sampling, numbers of pigs displaying clinical signs, applied antibiotic treatment, and number of sick and dead pigs from the start of the outbreak until the time of the interview.

#### Final visit (follow up)

During the final visit, second blood samples were collected from individually ear tagged pigs, and rectal temperature measured in the same pigs.

### Sample handling and diagnostics

Procedures for sample handling are presented in Fig. 1.

#### Pathological examination

Organs from 52 pigs were subject to post-mortem examination. The pericardium, pleura, trachea, bronchi, lung parenchyma and tracheobronchial lymph nodes from 28 to 24 pigs from outbreak herds and non–outbreak herds respectively, were examined at the Norwegian Veterinary Institute (NVI) according to a standardized protocol (Additional file 1). Tissue samples from the lungs, pleura and lymph nodes were fixed, processed, sectioned and stained for histological examination (Additional file 2). In total, 112 histological sections from the outbreak herds and 36 sections from 3 non-outbreak herds were examined following a standardized protocol (Additional file 2).

#### Bacteriology

Sampling (on charcoal transport swabs) for bacterial cultivation was performed during postmortem examination of 52 lungs and pleurae, see details in Table 3. The lung surface was flamed and aseptically incised before swabbing of lung tissue. The swabs were cultivated as a part of the routine diagnostics at NVI (Additional file 3).

#### Serotyping by whole genome sequencing

Serovar identification of cultured *A. pleuropneumoniae* (n = 31 isolates) was performed on sequence data, generated through whole genome sequencing of the *A. pleuropneumoniae* isolates at Statens Serum Institut (SSI), Copenhagen, Denmark. The serovar was determined based on the presence of the serovar specific cps operons [29, 30]. Details regarding the method are described in Additional file 3.

#### Serology

The serum samples (n = 282) were analyzed using commercial diagnostic kits for antibodies to *A. pleuropneumoniae*, influenza A virus, PRRSV, PRCV and *M. hyopneumoniae.* The analyses were performed as described by the manufacturers; details are given in Additional file 3. Interpretation of the test results were categorical, based on the cut-off values recommended by the test manufacturers. Presence of antibodies to PRRSV, porcine respiratory corona virus (PRCV) and *M. hyopneumoniae* were tested in the second serum sample (n = 141). Serum ELISA was conducted on paired serum samples (n = 282) from individual pigs for antibodies to influenza A virus and *A. pleuropneumoniae.*

#### Virology

The presence of influenza A virus and porcine circovirus type 2 (PCV2) nucleic acids in pooled oral fluids (n = 14) were analyzed with real time polymerase chain reaction (PCR) by in-house procedures (Additional file 3). A cycle threshold (Ct) value for influenza virus below 37 was considered positive. PCV2 quantitative PCR (qPCR) is a quantitative test where results are given as measured nucleotide copies in 200 µL sample, calculated from repeated measures at different Ct values and results are reported as low (< 10^4^ copies), moderate (10^4^–10^7^ copies) or high (> 10^7^ copies).

### Statistical analyses

Our sample size of 10 serum samples per herd was chosen based on an estimate of at least one positive animal if the prevalence of our disease in question is around 25% at a 95% confidence level. The same sample size was used for agents not present in the population, that we did not expect to find, due to practical reasons.

Statistical analyses of the data were performed using the software Stata (STATA SE/15 for Windows; Stata Corp., College Station, TX, USA). Descriptive numeric results are presented as average values and the standard deviation (SD) for data with a normal distribution, or median value followed by the interquartile range (iqr) for data that was not normally distributed.

Rectal temperatures from the first visit and from the final visit to the herd were compared. The variable “fever” was defined as a rectal temperature above 39.5 °C. Odds ratios for fever during the outbreak sampling compared to fever during follow-up visits, were calculated using a Stata 15 case–control odds-ratio calculator.

Morbidity was measured as the proportion of pigs with clinical signs of respiratory disease of the total number of pigs in the herd (herd morbidity) and in the compartment (compartment morbidity). Mortality was measured as the proportion of pigs dying during the outbreak, out of the total number of pigs in the herd (herd mortality) and in the compartment (compartment mortality). Case fatality, an indicator of pathogen virulence and disease lethality, was measured as the proportion of pigs that died during the outbreak and displayed clinical signs of respiratory disease prior to their death, out of the total number of pigs displaying respiratory disease.

A herd was classified as seroconverted if at least one pig shifted from negative to positive status and no pigs shifted from positive to negative status. The proportion of seroconverted pigs in each herd was calculated. Samples from pigs that could not be identified by ear tags (one herd, n = 10) were excluded. When calculating the incidence proportion and risk ratios for seroconversion to *A. pleuropneumoniae*, pigs that were seropositive on the first serum sample were excluded from the population at risk. Incidence proportion was defined as the proportion of the seronegative pigs that seroconvert during the time at risk. Time at risk was defined as time between paired serum samples. The risk ratio (RR) for a pig to seroconvert in outbreak herds, compared to non-outbreak herds, was calculated using a Stata15 Cohort study risk-ratio calculator the 95% confidence interval (CI). The statistical significance of the calculated association, whether it was likely that the RR was different from 1, was indicated by the reported *p* value.

## Results

### Clinical findings

Median number of days from the farmers noticed clinical signs of respiratory disease until calling the local veterinary practitioner was 1 day. Onset of outbreak was 35 days (median, iqr 43) after the pigs arrived at the compartment. The severity of the clinical signs varied between outbreak herds. Clinical signs reported by the veterinary practitioner were mainly sudden deaths (four herds) and dyspnea (three herds). Signs such as fever, bloody froth from oronasal openings, cough and lethargy were also reported, and it was observed that sick pigs were reluctant to chew on the cotton ropes used for OF sampling.

In all herds, intramuscularly administrated procaine benzylpenicillin was used to treat sick pigs over 3 to 5 days. In one herd, tiamulin was additionally administered in the drinking water for 4 days. Treatments were started by the veterinary practitioner during the first visit after the outbreak of disease. All herd owners reported the treatment to effectively reduce acute clinical signs and stop the further spread of disease.

The average compartment morbidity during the outbreak was 60% (SD 43, range 6–100%), while herd morbidity was 25% (SD 19, range 0.9–51%) in the outbreak herds. Case fatality rate during the disease outbreaks was on average 9% (SD 12, range 0–34%) over 5 days, suggestive of a low virulent agent. During the outbreaks, compartment mortality was 4% on average (SD 3, range 0–10%), while herd mortality was 2% (SD 2, range 0–5%). Proportion of pigs in the outbreak herds measuring a rectal temperature above 39.5 °C was 57.6% (n = 54) and 30% at the first and final visit, respectively. For the non-outbreak herds the proportion of pigs with a rectal temperature above 39.5 °C was 42.4% (n = 50) and 10% at first and final visit, respectively. The odds for a temperature above 39.5 °C were higher (odds ratio = 2.8, 95% CI 1.17–6.70), during outbreak than during follow-up in the outbreak herds. There were no dropouts among the study animals, the number of animals tested at the visits was the same. Median number of days between first and final visit was 22 days (iqr 5) in outbreak herds and 18 days (iqr 4) in non-outbreak herds.

### Diagnostics

#### Pathological examination

**Fig. 2:**
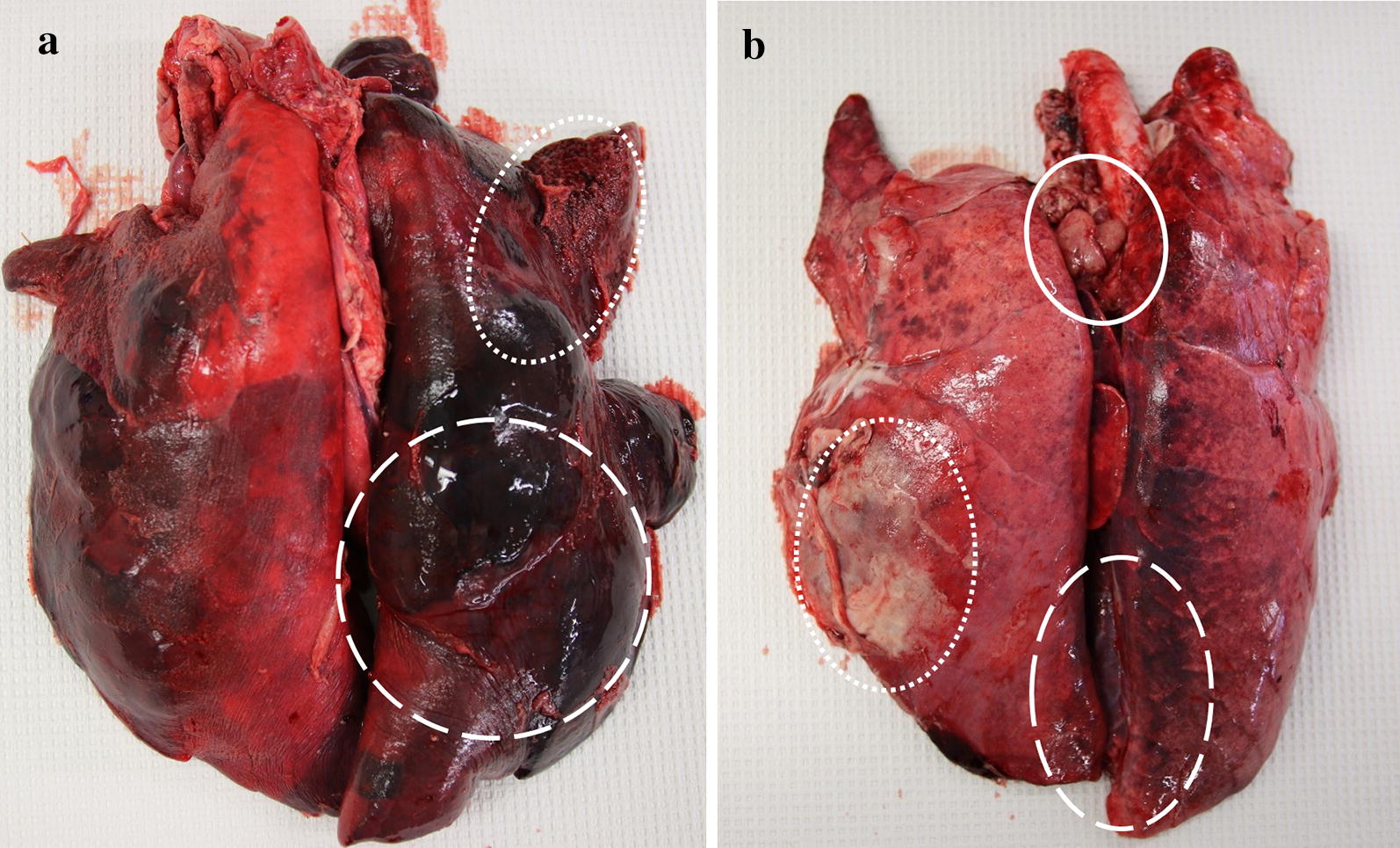
Lungs from pigs that displayed clinical signs of respiratory disease prior to death. Macroscopic lung lesions. **a.** Acute pleuropneumonia—Macroscopic lesions include fibrinopurulent pleuritis (dotted ring), edematous lungs with multifocal to coalescing hemorrhagic and hyperemic areas (dashed ring). *A. pleuropneumoniae* was cultured in abundance from pleura, left and right caudal lung lobes. **b** Chronic pleuritis, acute pneumonia. Macroscopic lesions include fibrous pleuritis (dotted ring), and focal, dark red areas of condensed lung tissue (dashed ring). The tracheobronchial lymph nodes were moderately enlarged (continuous ring). *A. pleuropneumoniae* was cultured from the right caudal lung lobe; no bacteria were retrieved from the chronic pleuritic lesion

Results from the pathological examinations of 52 organs are presented on herd level in Table 2. Gross pathology of the lungs was detected in all pigs (n = 28) from the outbreak herds. Acute pleural lesions were reported in 25 of these pigs (89%) and chronic pleural lesions, were found in one. Typical lesions of acute pneumonia were found in all the pigs. The acute lesions were principally dorsally distributed in all lung lobes, but the caudal lobes were the most affected. Chronic lung lesions were observed in one pig. Moderate to severe enlargement of the tracheobronchial lymph nodes was a prevalent finding (n = 22, 73%) in the pigs with pneumonia. Characteristic gross lung lesions are shown in Fig. 2.

**Table 2:** Results from gross pathology, bacteriology, serology and virology from seven outbreak herds (from 1 to 7) and seven non-outbreak herds (from 8 to 14)

Herd no.	Number of samples	Pleura gross pathology No. of pigs		Lung gross pathology No. of pigs		Bacteriology^a^ No. of pigs (%)		Serology No. of pigs (%)		Virology RT-qPCR on pooled OF sample	
	Lungs/serum/OF	Acute lesion^b^	Chronic lesion^c^	Acute lesion^d^	Chronic lesion^e^	APP culture^f^		APP seroconver-sion^g^	SIV sero-conversion	PCV2 detection^h^ (category)	SIV detection
						Pleura	Lung				
1	5/10/1	5	–	5	–	4 (80)	5 (100)	3 (30)	–	18,000 (mod)	–
2	3/10/1	3	–	3	1^i^	3 (100)	3 (100)	9 (90)	1(10)^j^	9700 (low)	–
3	5/10/1	4	1	5	–	5 (100)	5 (100)	6 (60)	–	1500 (low)	–
4	3/10/1	2	–	3	–	2 (67)	3 (100)	5 (50)	–	< 10 (low)	–
5	4/10/1	4	–	4	–	4 (100)	4 (100)	10 (100)	–	19,000 (mod)	–
6	5/10/1	5	–	5	–	5 (100)	5 (100)	6 (60)	–	55,000 (mod)	–
7	3/10/1	2	1	3	–	3 (100)	3 (100)	–^k^	–	602 (low)	–
8	3/10/1	1	–	–	–	0	0	4 (40)	–	58,000 (mod)	–
9	3/10/1	–	–	1	1	0	1 (33)	0	–	16,000 (mod)	–
10	4/10/1	–	1	–	–	0	0	2 (20)	–	220,000 (mod)	–
11	4/10/1	–	–	–	–	0	0	1 (10)	–	430,000 (mod)	–
12	3/10/1	–	–	–	–	0	0	8 (80)	–	850,000 (mod)	–
13	4/10/1	–	–	–	–	0	0	4 (40)	–	15,000 (mod)	–
14	3/10/1	–	–	–	1	0	1 (33)	1 (10)	–	5270 (low)	–

OF, oral fluids; APP, *Actinobacillus pleuropneumoniae;* PCV2, Porcine Circovirus type 2; PCR, reverse transcription quantitative polymerase chain reaction; SIV, Swine influenza virus; mod, moderate virus concentration

^a^Swabs from lesions on pleura and lung tissue in right and left, cranial and caudal lobe. If there were no macro-pathological lesions, swabs were collected from pleura, left cranial lobe and right caudal lobe, respectively

^b^Fibrinopurulent pleuritis

^c^Fibrous pleuritis

^d^Demarcated, firm, deep red lung tissue with hemorrhage

^e^Chronic necrotic areas and abscess formation

^f^Growing on lung, pleura and/or pericardium

^g^Proportion of total sample size, in most herds there were seropositive individuals at first sampling, leaving the true population at risk smaller

^h^DNA copies per 200 uL

^i^Acute lesion also present

^j^In this herd, 2 (20%) pigs had a decreasing serum level of SIV antibodies

^k^Unable to identify individual pigs at second sample due to missing unique identifiers

In the non-outbreak herds various gross lung lesions were detected in seven of the 24 pigs (29%). Pleuritis was observed in two of 24 pigs (8%), where one had an acute pleuritis, and the second pig focal chronic pleuritis. Pneumonia was observed in four other pigs. Mild, focal, acute lesions were seen in two of them, while similar acute lesions and abscess formation was seen in another. Multifocal, necrotizing, chronic pneumonia was diagnosed in the fourth pig. A single pig from a non-outbreak herd had gross lung lesions of multifocal bleeding and mottled grayish green areas indicative of larval migration by *Ascaris suum.* Diagnostic results for individual herds, including the gross findings are summarized in Table 2.

Histopathological changes agreed with the acute macroscopic lesions observed. Histological examination revealed fibrin and neutrophil deposits on the pleura. In the lung parenchyma there was alveolar filling with necrotic leukocytes, neutrophils and fibrin. Interstitial edema and hemorrhage, peribronchial and peribronchiolar leukocyte infiltration was observed. Subacute to chronic, necrotic lesions of varying sizes were demarcated by macrophages, lymphocytes and plasma cells surrounded by connective tissue. Histopathological pathological changes in lymph nodes included neutrophils in subcapsular sinuses in cases of acute pneumonia. When investigating samples from lungs without gross lesions from the non-outbreak herds there was occasional occurrence of mononuclear cell infiltrates and fibrin deposits on the pleura or in alveolar lumen, and areas of interstitial bleeding.

#### Bacteriology

*Actinobacillus pleuropneumoniae* was cultured from all sampled pigs (n = 28) from outbreak herds (n = 7). Abundant growth of *A. pleuropneumoniae* was present in lung tissue in all 28 pigs and on pleura in 26 pigs. In samples from 20 of the lungs and 13 pleurae, *A. pleuropneumoniae* was the sole microbial species detected. In the remaining samples, a range of bacteria were detected in addition to *A. pleuropneumoniae* and the results are shown in Table 3. Swabs from non-outbreak pigs’ lungs produced mostly negative bacteriology. From non-outbreak herds, *A. pleuropneumoniae* was isolated from lung parenchyma in two out of 24 pigs. The *A. pleuropneumoniae* isolates originated mainly from areas with acute gross pathology (Table 2). In one non-outbreak pig *A. pleuropneumoniae* was cultured from a chronic lung lesion.

**Table 3:** Summary of bacteriologic findings on at least one sample from lung and pleura in 28 pigs from outbreak herds and 24 pigs from non-outbreak herds

Microbial species	Outbreak herds^a^		Non-outbreak herds^a^	
	Lung n = 28	Pleura n = 28	Lung n = 24	Pleura n = 24
*Actinobacillus pleuropneumoniae*	28^b^	26^c^	2^d^	0
*Pasteurella multocida*	1	3	–	–
*Streptococcus* spp.	5	10	–	–
*Haemophilus parasuis*	–	–	–	–
*Bordetella bronchiseptica*	–	–	1	–
Coliform bacteria	2	2	–	–
Other: *Actinomyces hyovaginalis, Trueperella pyogenes, Proteus mirabilis, Fusobacterium* spp.	3	–	1	–
Unspecific mixed culture	–	1	4	1

^a^In total, 28 pleural swabs and 52 lung swabs were collected in outbreak herds, while 24 pleural and 49 lung swabs were collected in non-outbreak herds. The sum of findings per lung or pleura are presented here

^b^From swabs of acute lung lesions. *A. pleuropneumoniae* was the sole microbial species detected in lung samples from 20 of these pigs

^c^From swabs of pleuritic lesions. *A. pleuropneumoniae* was cultured from 26 pleuritic lesions. *A. pleuropneumoniae* was the sole microbial species detected in pleura samples from 13 of these pigs

^d^From swabs of lung lesions. *A. pleuropneumoniae* was the sole microbial species detected in one lung with chronic lesions, while the other was a mixed culture from an acute lesion

Serotyping of *A. pleuropneumoniae* on genome level revealed that all sampled isolates belonged to serovar 8.

#### Serology

The 282 serum samples were successfully analyzed in one session. Antibodies to *A. pleuropneumoniae* were detected in samples from six (86%) outbreak herds and four (57%) non outbreak herds. At the first serum sample, 35% (25 of 71) of the pigs in the outbreak herds were seropositive, and 37% (26 of 70) in the non-outbreak herds. At the second serum sample, 89% (63 of 71) and 60% (42 of 70) of the pigs were positive in the outbreak and non-outbreak herds respectively, details are listed in Table 2. Six outbreak herds and six non-outbreak herds were considered seroconverted, indicative of an active infection in the period from the first to the second visit. Seroconversion in the seventh outbreak herd could not be assessed due to missing ear tags. Proportion of seroconverted pigs in each outbreak herd ranged from 30 to 100%, and from 0 to 80% in non-outbreak herds (Table 2). Incidence proportion was 0.96 (SD 0.10) in outbreak herds over the median time at risk of 22 days. Incidence proportion in the non-outbreak herds was 0.44 (SD 0.36) over the median time at risk of 18 days. The risk for seroconversion was more than double compared to pigs from non-outbreak herds (RR 2.3 [1.50–3.43 95% CI; P < 0.001]).

Antibodies to influenza A virus were detected in one outbreak herd, where one pig seroconverted during the sampling period, and two pigs were found to have a reduced antibody titer to below cutoff. Influenza A-antibodies were not detected in the remaining six outbreak herds or the non-outbreak herds. The proportion of SIV seropositive herds was 7% out of the herds combined. Antibodies to *M. hyopneumoniae*, PRCV and PRRSV were not detected in samples from any herds.

#### Virology

The 14 pooled OF samples from 28 pens, median number of pigs per pen was 10 (range 5–19), were all negative for Influenza A Viruses. Quantification of PCV2 by RT-qPCR turned out low or moderate in all samples, results per herd are shown in Table 2.

## Discussion

Field outbreaks of acute respiratory disease in Norwegian fattening pigs were investigated and *A. pleuropneumoniae* serovar 8 was the main pathogen detected, with negligible presence of co-infections. Clinical signs reported were in agreement with previous reports of *A. pleuropneumoniae* infections, which are described to have a diverse clinical presentation [31]. Even with the large variation in morbidity and mortality rates, the results from this study were in line with observations from other studies, as research on outbreak characteristics of respiratory disease show that morbidity can range from 10 to 100% [26]. Mortality during outbreaks of acute porcine pleuropneumonia is usually reported to be between 1 and 10% [26]. Case fatality rates are not commonly included in this research literature but is a more precise measure of the lethality of a disease, especially if little information about other illnesses is available. Disease that affects mortality are likely to have common risk factors [32] and the use of case fatality rate is a more robust measurement and less subjected to confounders such as that of other illnesses.

Even as a single infectious primary agent, *A. pleuropneumoniae* can cause severe clinical signs. During acute porcine pleuropneumonia, high fever is common [33, 34]. For pigs in the age range from 3 to 6 months, body temperatures normally span from 38.5 to 39.3 °C [35], and the proportion of pigs displaying a fever can be indicative of an outbreak. In the present study, the pigs were restrained by snaring the upper jaw during clinical examination and blood collection, which is stressful for the animal [36]. The cutoff for fever at 39.3 °C + 0.2 was used in the study to compensate for this stress. Higher odds for displaying fever in the herds during outbreak than at the final visit were found among the pigs in this study. This signified body temperature as a disease characteristic during outbreaks of porcine pleuropneumonia, although technical biases like personnel and thermometers used might have influenced our results. This coincided with results from a recent study from Finland [37].

There are 18 acknowledged *A. pleuropneumoniae* serovars, of which some were recently described [38]. From the Norwegian pig population, serovars 2, 6, 7, 8 and 10 have previously been reported [39]. Serovar 8 has been most commonly associated with clinical disease in recent years, followed by type 6 [40]. However, these previous findings were all based on antibody agglutination tests which are prone to cross-reactions, for instance between serovars 3, 6, 8 and 15 [41]. All *A. pleuropneumoniae* strains in this study belonged to type 8, raising questions about the importance of serovar 6. Underestimation of serovar 8 has occurred in Canada [42], England and Wales [43]. Serovar 8 is typically viewed as low virulent and is less often associated with clinical disease globally. In a study describing clinical presentation of different serovars in experimentally infected pigs [33], serovars that were less commonly associated with disease were able to produce severe clinical signs, including high fever. This could perhaps be a result of absence of other respiratory agents including more virulent serovars of *A. pleuropneumoniae*.

The macro- and histopathologic findings were typical for acute pleuropneumonia caused by *A. pleuropneumoniae* [44–46], supporting that *A. pleuropneumoniae* was the main etiologic pathogen in these outbreaks. Direct agent detection, primarily by bacteriological culturing in affected lung tissue obtained during necropsy, is considered the most adequate method for diagnosing porcine pleuropneumonia [31]. Direct PCR is a method that would be expected to yield similar results but would not allow for storing of the bacterial isolates for further molecular testing, as was done in this study. We observed a low incidence of pathological lesions in non-outbreak herds, and *A. pleuropneumoniae* was only isolated from lesions resembling porcine pleuropneumonia. Other bacteria, including *P. multocida* and *Streptococcus* spp., were also detected in a few samples in this study. Both are known opportunistic bacteria that colonize the upper respiratory tract of healthy pigs [4]. *Streptococcus suis* is the most important streptococcal swine pathogen found to contribute to bronchopneumonia [47]. It is not unlikely that the bacteria could colonize areas already infected with *A. pleuropneumoniae*. The lesions might then be hard to distinguish from the primary pathogen, particularly if large parts of the lungs are affected. In one outbreak herd all five lungs had growth of other bacteria. They could have been contaminated during collection, transport or sampling. Alternatively, these pigs were all colonized by secondary bacterial pathogens. The number of herds included in this study was too low to investigate whether the presence of these bacteria was linked to any differences in outbreak characteristics or diagnostic results. The low occurrence of common secondary invaders could have been explained by the short time span between registered disease and sampling. It has been questioned whether the actions that led to the eradication of *M. hyopneumoniae* from the Norwegian pig population [18] also significantly reduced the occurrence of other pathogens. This has not yet been investigated.

Treatment with procaine benzylpenicillin was in line with the therapeutic guidelines published by the Norwegian Medicines Agency as the drug of choice for acute porcine pleuropneumonia [48]. Similar recommendations have been published in Finland and Sweden [49, 50]. In Denmark, tilmicosin and tulathromycin have been commonly used against acute pleuropneumonia [51] partly due to the convenience of peroral administration, not due to reduced susceptibility to benzylpenicillin. National surveillance programs for antimicrobial resistance in these countries have recently reported a high proportion of *A. pleuropneumoniae* isolates being susceptible to benzylpenicillin [51–53]. Nevertheless, there are no recently published studies on the efficacy of procaine benzylpenicillin for porcine pleuropneumonia in Norway. Such knowledge of causative pathogens is the fundament for correct and prudent use of antimicrobials. The details to antimicrobial resistance patterns of *A. pleuropneumoniae* in Norway are currently being studied further.

Seroconversion to *A. pleuropneumoniae* had occurred in most of the herds, in many cases in absence of clinical disease. The risk for seroconversion to *A. pleuropneumoniae* for pigs in outbreak herds was more than double compared to pigs from non-outbreak herds, despite small within-herd populations at risk due to many seropositive pigs in the first serum samples. Seroconversion to less virulent strains might have happened without resulting in a cross-protection to the outbreak-causing serovar. In Finland, *Haimi*-*Hakala* et al. observed no difference in either prevalence of seroconverted herds or proportion of seroconverted pigs per herd in the outbreak case group and non-outbreak control group [37]. They discuss that neither single or paired serum sampling for the diagnosis of acute respiratory disease in field conditions is of much value due to both a lack of details concerning the initiation time of infection and a high prevalence of subclinical infections with *A. pleuropneumoniae*. The risk for seroconversion was not addressed in their paper. A Danish study from 2004 investigated correlations in seroconversion to *A. pleuropneumoniae* and concluded that variation in seroconversion was mainly explained by a common batch level factor, that varies between farms and batches within a farm [54]. Outbreaks of disease might be viewed as a batch level factor in this sense. In cases of all-in-all-out rearing by compartment, which is common, batches of pigs are usually housed separately. As we observed, the outbreaks were often restricted to single compartments. Risk factors can be related to animal housing, management and environment [26], and infection pressure might be increased during clinical disease and is a likely trigger for seroconversion. Risk factor analyses were beyond the scope of this paper due to a lower number of herds in our study than what was expected. The seeming decrease in outbreak occurrence might have resulted from of a collective effort in the Norwegian pig production system to increase the health status of herds with reoccurring problems with respiratory disease prior to our sampling.

When investigating SIV antibody titers we found that only one outbreak herd was seropositive. Even though one pig seroconverted during the sampling period, two pigs were found to have reduced antibody titer. Since a single false-positive serological reactor could not be excluded, the true status of these animals was uncertain. There being multiple false-positive reactions in one herd, which would have been the case here, was perhaps less likely. The proportion of seropositive herds in this study was less than what is found on a national level, where approximately 25% of the herds are reported positive [21]. The virology results from our study suggested that neither SIV nor PCV2 contributed to the disease outbreaks in the study population. The absence of SIV in all OF samples supported the lack of pathological lesions and serological results indicative of SIV infection. No difference was detected in PCV2 levels between the outbreak- and the non-outbreak herds. Reluctancy of sick pigs to chew on the ropes could have resulted in unrepresentative PCV2 levels. Since PCV2 levels was tested on pooled samples we have no information on the individual pig’s contribution to the sample.

The health status of the Norwegian pig population is very good and have many similarities to the one of Finland in the sense that they are free from *M. hyopneumoniae,* PRRSV and until recently PRCV [21]. In Finland, a more diverse outbreak etiology has been observed [37]. In the Finnish study, *A. pleuropneumoniae* was found to be the most likely cause of disease in 14 of the 20 sampled herds. In most of these herds, *A. pleuropneumoniae* was the only etiologic pathogen identified. Similarly, 16 outbreaks of respiratory disease were studied in the Netherlands [10] concluding that five of these were most likely caused by *A. pleuropneumoniae*, while seven were caused by SIV (H1N1) and (H3N2). Like in our study, they did not find any clear evidence of specific dual infections.

## Conclusion

The main etiological pathogen of acute outbreaks of respiratory disease in the included Norwegian fattening pigs was *A. pleuropneumoniae*. All pigs from outbreak herds were found to have typical lesions of acute porcine pleuropneumonia, and only *A. pleuropneumoniae* serovar 8 was identified. The clinical presentation and pathology of *A. pleuropneumoniae* was in line with previous reports on field outbreaks internationally. Co-infections did not seem to be of impact on disease development.

## Supplementary information

**Additional file 1.** Protocol for postmortem sampling. A scheme for a standardized postmortem evaluation and sampling of pigs’ lungs. The scheme was compiled at the pathology department at The Norwegian Veterinary Institute to be used in the study of acute respiratory disease outbreaks.

**Additional file 2.** Histology protocol. A scheme for a standardized histologic evaluation of sections from pigs’ lungs, pleura and tracheobronchial lymph nodes, including a description of section preparation. The scheme was compiled by members of the project group Grisefine lunger to be used in the study of acute respiratory disease outbreaks.

**Additional file 3.** Details of sample handling and diagnostics. A document containing extended details of sample handling and laboratory diagnostic methods performed in the study of acute respiratory disease outbreaks.

## Data Availability

The datasets used and/or analyzed during the current study are available from the corresponding author on reasonable request.

## References

[1] van Alstine WG, Zimmermann JJ, Karriker LA, Ramirez A, Schwartz KJ, Stevenson GW (2012). Respiratory System. Diseases of Swine.

[2] Straw BE, Shin SJ, Yeager AE (1990). Effect of pneumonia on growth rate and feed efficiency of minimal disease pigs exposed to *Actinobacillus pleuropneumoniae* and *Mycoplasma hyopneumoniae*. Prev Vet Med..

[3] Jager HJ, McKinley TJ, Wood JL, Pearce GP, Williamson SM, Woodger N, et al. A tool to assess the economic impact of pleurisy in slaughter pigs. In: Proceedings of the 21^st^ International Pig Veterinary Society Congress; 2010 July 18-21; Vancouver, Canada. http://www.theipvs.com/wp-content/uploads/2019/07/IPVS-2010-Final-Proceedings.pdf.

[4] Brockmeier SL, Halbur PG, Thacker EL, Brogden KA, Guthmiller JM (2002). Porcine Respiratory Disease Complex. Polymicrobial Diseases.

[5] Palzer A, Ritzmann M, Wolf G, Heinritzi K (2008). Associations between pathogens in healthy pigs and pigs with pneumonia. Vet Rec..

[6] Thacker EL, Halbur PG, Ross RF, Thanawongnuwech R, Thacker BJ (1999). *Mycoplasma hyopneumoniae* potentiation of porcine reproductive and respiratory syndrome virus-induced pneumonia. J Clin Microbiol.

[7] van Dixhoorn IDE, Reimert I, Middelkoop J, Bolhuis JE, Wisselink HJ, Koerkamp PWGG (2016). Enriched housing reduces disease susceptibility to co-infection with Porcine Reproductive and Respiratory Virus (PRRSV) and *Actinobacillus pleuropneumoniae* (*A. pleuropneumoniae*) in young pigs. PLoS ONE.

[8] Loving CL, Brockmeier SL, Vincent AL, Palmer MV, Sacco RE, Nicholson TL (2010). Influenza virus coinfection with *Bordetella bronchiseptica* enhances bacterial colonization and host responses exacerbating pulmonary lesions. Microb Pathog.

[9] Bochev I (2007). Porcine respiratory disease complex (PRDC): a review I Etiology, epidemiology, clinical forms and pathoanatomical features. Bulg J Vet Med..

[10] Loeffen WLA, Kamp EM, Stockhofe-Zurwieden N, van Nieuwstadt APKMI, Bongers JH, Hunneman WA (1999). Survey of infectious agents involved in acute respiratory disease in finishing pigs. Vet Rec.

[11] Pomorska-Mól M, Dors A, Kwit K, Kowalczyk A, Stasiak E, Pejsak Z (2017). Kinetics of single and dual infection of pigs with swine influenza virus and *Actinobacillus pleuropneumoniae*. Vet Microbiol.

[12] Thacker EL, Thacker BJ, Janke BH (2001). Interaction between *Mycoplasma hyopneumoniae* and swine influenza virus. J Clin Microbiol.

[13] Hansen MS, Pors SE, Jensen HE, Bille-Hansen V, Bisgaard M, Flachs EM (2010). An investigation of the pathology and pathogens associated with porcine respiratory disease complex in Denmark. J Comp Pathol.

[14] Kim J, Chung HK, Chae C (2003). Association of porcine circovirus 2 with porcine respiratory disease complex. Vet J..

[15] KOORIMP, KIF. Annual report 2018 KOORIMP and KIF [In Norwegian]. Animalia. 2019. https://www.animalia.no/contentassets/7b27e28ef6bf4e878416cc6664a440e1/koorimp-arsmelding-2018-web.pdf. Accessed 12 Des 2019.

[16] Regulation on swine and poultry production [in Norwegian]. FOR-2004-04-01-611 (2004). Available from: https://lovdata.no/dokument/SF/forskrift/2004-04-01-611.

[17] Animalia. Annual statistical report 2018 [in Norwegian]. 2019. https://www.animalia.no/contentassets/28e0db72674d496186f0570a9e606fca/arsstatistikk-2018.pdf. Accessed 01 Nov 2019.

[18] Gulliksen SM, Lium B, Framstad T, Jørgensen A, Skomsøy A, Gjestvang M, et al. The successful eradication of *Mycoplasma hyopneumoniae* from Norwegian pig herds – 10 years later. In: Abstract book of the 11^th^ European Symposium of Porcine Health Management; 2019 May 22-24; Utrecth, The Netherlands. https://www.esphm2019.org/download/2569/.

[19] Animalia. The Norwegian livestock industry´s joint action plan on antimicrobial resistance. 2017. https://www.animalia.no/contentassets/05c57591f69d4e1da9bb5c44668bd0c1/eng_husdyrnaringas-hplan-amr-endelig-enkeltsider_220617.pdf. Accessed 16 Sept 2019.

[20] Grøntvedt CA, Er C, Gjerset B, Hauge AG, Brun E, Jørgensen A (2013). Influenza A(H1N1)pdm09 virus infection in Norwegian swine herds 2009/10: the risk of human to swine transmission. Prev Vet Med..

[21] Grøntvedt CA, Nordstoga AB, Jonsson ME. The surveillance programme for specific viral infections in swine herds in Norway 2018. The Norwegian Veterinary Institute. 2019. https://www.vetinst.no/en/surveillance-programmes/viral-infections-in-swine/_/attachment/download/e5793dca-57d2-41eb-bd34-9a99e5d11b98:ef9892272432bafa4b61bc30ae1130952606075b/2019_OK_Viral%20infections%20in%20swine_Report%202018.pdf. Accessed 07 Nov 2019.

[22] Er C, Lium B, Tavornpanich S, Hofmo PO, Forberg H, Germundsson Hauge A (2014). Adverse effects of Influenza A(H1N1)pdm09 virus infection on growth performance of Norwegian pigs—a longitudinal study at a boar testing station. BMC Vet Res..

[23] Grøntvedt CA. In: The Norwegian Veterinary Institutes activity report 2016 [in Norwegian]. The Norwegian Veterinary Institute. 2017. Available from: https://www.vetinst.no/rapporter-og-publikasjoner/rapporter/2017/veterinaerinstituttets-faglige-aktivitetsrapport/_/attachment/download/ae016944-69b3-46f1-924d-8df8f82eca8e:94edd0c45b069ed56bfbb15e35c82059556bb988/2017_20_Veterin%C3%A6rinstituttets%20faglige%20aktivitetsrapport%202016%20-%20komplett.pdf. p. 12-13. Accessed 16 Sep 2019.

[24] Vigre H, Angen O, Barfod K, Lavritsen DT, Sorensen V (2002). Transmission of *Actinobacillus pleuropneumoniae* in pigs under field-like conditions: emphasis on tonsillar colonisation and passively acquired colostral antibodies. Vet Microbiol.

[25] Chiers K, Donne E, Van Overbeke I, Ducatelle R, Haesebrouck F (2002). *Actinobacillus pleuropneumoniae* infections in closed swine herds: infection patterns and serological profiles. Vet Microbiol.

[26] Klinkenberg D, Tobias TJ, Bouma A, van Leengoed LA, Stegeman JA (2014). Simulation study of the mechanisms underlying outbreaks of clinical disease caused by *Actinobacillus pleuropneumoniae* in finishing pigs. Vet J..

[27] Gulliksen SM (2016). Respiratory disease in pigs—an increasing problem? [in Norwegian]. Go’mørning..

[28] Prickett JR, Kim W, Simer R (2008). Oral-fluid samples for surveillance of commercial growing pigs for porcine reproductive and respiratory syndrome virus and porcine circovirus type 2 infections. J Swine Health Prod.

[29] Bosse JT, Li Y, Fernandez Crespo R, Lacouture S, Gottschalk M, Sarkozi R (2018). Comparative sequence analysis of the capsular polysaccharide loci of *Actinobacillus pleuropneumoniae* serovars 1-18, and development of two multiplex PCRs for comprehensive capsule typing. Vet Microbiol.

[30] Bosse JT, Chaudhuri RR, Li Y, Leanse LG, Fernandez Crespo R, Coupland P (2016). Complete Genome Sequence of MIDG2331, a Genetically Tractable Serovar 8 Clinical Isolate of *Actinobacillus pleuropneumoniae*. Genome Announc..

[31] Sassu EL, Bosse JT, Tobias TJ, Gottschalk M, Langford PR, Hennig-Pauka I (2017). Update on *Actinobacillus pleuropneumoniae*-knowledge, gaps and challenges. Transbound Emerg Dis..

[32] Maes DG, Duchateau L, Larriestra A, Deen J, Morrison RB, de Kruif A (2004). Risk factors for mortality in grow-finishing pigs in Belgium. J Vet Med B Infect Dis Vet Public Health..

[33] Costa G, Oliveira S, Torrison J, Dee S (2011). Evaluation of *Actinobacillus pleuropneumoniae* diagnostic tests using samples derived from experimentally infected pigs. Vet Microbiol.

[34] Tobias TJ, Bouma A, Klinkenberg D, Daemen AJ, Stegeman JA, Wagenaar JA (2012). Detection of *Actinobacillus pleuropneumoniae* in pigs by real-time quantitative PCR for the apxIVA gene. Vet J..

[35] Dewey CE, Straw BE, Zimmermann JJ, Karriker LA, Ramirez A, Schwartz KJ, Stevenson GW, Zhang J (2019). Herd evaluation. Diseases of Swine.

[36] Parrott RF, Lloyd DM (1995). Restraint, but not frustration, induces prostaglandin-mediated hyperthermia in pigs. Physiol Behav.

[37] Haimi-Hakala M, Halli O, Laurila T, Raunio-Saarnisto M, Nokireki T, Laine T (2017). Etiology of acute respiratory disease in fattening pigs in Finland. Porcine Health Manag..

[38] Bosse JT, Li Y, Sarkozi R, Fodor L, Lacouture S, Gottschalk M (2018). Proposal of serovars 17 and 18 of *Actinobacillus pleuropneumoniae* based on serological and genotypic analysis. Vet Microbiol.

[39] Falk K, Lium BM, Ødegaard Ø. Occurrence of lung lesions and antibodies to serotypes 2 and 6 of *Actinobacillus pleuropneumoniae* and *Haemophilus parasuis* in 5176 slaughter weight pigs from 113 elite herds in Norway. In: Proceedings of the 11^th^ International Pig Veterinary Society Congress; 1990 July 1-5. Lusanne; Switzerland.

[40] Norwegian Veterinary Institute. Respiratory diseases [in Norwegian]. 2014. http://multiconsult.eurest.no/nor/Temasider/Svin/Fakta-om-svinesykdommer/LuftveislidelserMycoplasma-hyorhinis.html. Accessed 06 Aug 2019.

[41] Mittal KR, Higgins R, Lariviere S (1988). Quantitation of serotype-specific and cross-reacting group-specific antigens by coagglutination and immunodiffusion tests for differentiating *Actinobacillus* (*Haemophilus*) *pleuropneumoniae* strains belonging to cross-reacting serotypes 3, 6, and 8. J Clin Microbiol.

[42] Gottschalk M (2015). The challenge of detecting herds sub-clinically infected with *Actinobacillus pleuropneumoniae*. Vet J..

[43] Li Y, Bosse JT, Williamson SM, Maskell DJ, Tucker AW, Wren BW (2016). *Actinobacillus pleuropneumoniae* serovar 8 predominates in England and Wales. Vet Rec..

[44] Gottschalk M, Broes A, Zimmermann JJ, Karriker LA, Ramirez A, Schwartz KJ, Stevenson GW (2019). Actinobaccilosis. Diseases of Swine.

[45] Lium B, Falk K (1991). An abattoir survey of pneumonia and pleuritis in slaughter weight swine from 9 selected herds. I. Prevalence and morphological description of gross lung lesions. Acta Vet Scand.

[46] Falk K, Lium B (1991). An abattoir survey of pneumonia and pleuritis in slaughter weight swine from 9 selected herds. III. Serological findings and their relationship to pathomorphological and microbiological findings. Acta Vet Scand.

[47] Gottschalk M, Segura M, Zimmermann JJ, Karriker LA, Ramirez A, Schwartz KJ, Stevenson GW (2019). Streptococcosis. Diseases of Swine.

[48] Norwegian Medicines Agency. Therapy recommendations: Use of antimicrobial drugs in production animals [in Norwegian]. 2012. https://legemiddelverket.no/Documents/Veterin%C3%A6rmedisin/Terapianbefalinger/Terapianbefaling_bruk%20av%20antibakteriellt%20midler%20til%20produks.pdf. Accessed 12 Dec 2019.

[49] Evira, Finnish Food Safety Authority. Recommendations for the use of antimicrobials in the treatment of the most significant infectious and contagious diseases in animals. 2018. https://www.ruokavirasto.fi/globalassets/viljelijat/elaintenpito/elainten-laakitseminen/hallittu_laakekekaytto/mikrobilaakekaytonperiaatteet/mikrobilaakkeiden_kayttosuositukset_en.pdf. Accessed 12 Dec 2019.

[50] Swedish medical products agency. Antimicrobial drug dosage for pigs—new recommendations [in Swedish]. Information från Läkemedelsverket. 2012;23. Available from: https://lakemedelsverket.se/upload/halso-och-sjukvard/behandlingsrek-vet/Rek%20-%20dosering%20av%20antibiotika%20till%20gris%5b1%5d.pdf. Accessed 16 Sept 2019.

[51] Statens Serum Institut, National Food Institute. DANMAP 2018 - Use of antimicrobial agents and occurrence of antimicrobial resistance in bacteria from food animals, food and humans in Denmark 2019. 2019. https://www.danmap.org/-/media/arkiv/projekt-sites/danmap/danmap-reports/danmap-2018/danmap_2018.pdf?la=en. Accessed 20 Sept 2019.

[52] Nykäsenoja S, Olkkola S, Pohjanvirta T, Biström M, Kaartinen L, Helin-Soilevaara H, *et. al.* FINRES-Vet 2018: Finnish Veterinary Antimicrobial Resistance Monitoring and Consumption of Antimicrobial Agents. Finnish Food Authority publications. 2019;6. https://helda.helsinki.fi/bitstream/handle/10138/307519/finres_vet_2018_141119.pdf?sequence=1&isAllowed=y. Accessed 12 Dec 2019.

[53] Public Health Agency of Sweden and National Veterinary Institute. Swedres-Svarm 2017: Consumption of antibiotics and occurrence of resistance in Sweden. 2018. https://www.folkhalsomyndigheten.se/contentassets/2ec8ee5ab1674c75beec834ff903ec43/swedres-svarm-2017-18003.pdf. Accessed 12 Dec 2019.

[54] Vigre H, Dohoo IR, Stryhn H, Busch ME (2004). Intra-unit correlations in seroconversion to *Actinobacillus pleuropneumoniae* and *Mycoplasma hyopneumoniae* at different levels in Danish multi-site pig production facilities. Prev Vet Med..

