# Supplementary material for: A descriptive study of acute outbreaks of respiratory disease in Norwegian fattening pig herds
Source: Acta Vet Scand. 2020 Jun 24;62:35. doi: 10.1186/s13028-020-00529-z (PMC7312110; doi:10.1186/s13028-020-00529-z)
Supplement: Supplementary file 1 — Additional file 1. Protocol for postmortem sampling. A scheme for a standardized postmortem evaluation and sampling of pigs’ lungs. The scheme was compiled at the pathology department at The Norwegian Veterinary Institute to be used in the study of acute respiratory disease outbreaks. [file 13028_2020_529_MOESM1_ESM.pdf]

Protocol for postmortem sampling. *A scheme for a standardized post mortem evaluation and sampling of pigs' lungs. The scheme was compiled at the pathology department at The Norwegian Veterinary Institute to be used in the study of acute respiratory disease outbreaks.*

**Journal number**<br><br>**Case/control**	**Lung no.1**	**Lung no. 2**	**Lung no. 3**	**Lung no. 4**	**Lung no. 5**
**Pericarditis**<br>Type<br>+ sample for bacteriology (If no pleuritis)	0/ fibrin-pur/ fibrous<br>+ bact	0/ fibrin-pur/ fibrous<br>+ bact	0/ fibrin-pur/ fibrous<br>+ bact	0/ fibrin-pur/ fibrous<br>+ bact	0/ fibrin-pur/ fibrous<br>+ bact
**Pleuritis**<br>Type and distribution<br>+ sample for bacteriology	0 / fibrin-pur / fibrous<br>R cran-lobe/ caud-lobe<br>L cran-lobe / caud-lobe<br>+ bact	0 / fibrin-pur / fibrous<br>R cran-lobe/ caud-lobe<br>L cran-lobe / caud-lobe<br>+ bact	0 / fibrin-pur / fibrous<br>R cran-lobe/ caud-lobe<br>L cran-lobe / caud-lobe<br>+ bact	0 / fibrin-pur / fibrous<br>R cran-lobe/ caud-lobe<br>L cran-lobe / caud-lobe<br>+ bact	0 / fibrin-pur / fibrous<br>R cran-lobe/ caud-lobe<br>L cran-lobe / caud-lobe<br>+ bact
Take overview photo	Dorsal and ventral	Dorsal and ventral	Dorsal and ventral	Dorsal and ventral	Dorsal and ventral
**Tracheitis/bronchitis**<br>Type<br>+ sample for bacteriology	0 / fibrin -pur<br>+ bact	0 / fibrin-pur<br>+ bact	0 / fibrin-pur<br>+ bact	0 / fibrin-pur<br>+ bact	0 / fibrin-pur<br>+ bact
Tracheobronchial lymph nodes	0 / mod enlarged / severely enlarged	0 / mod enlarged / severely enlarged	0 / mod enlarged / severely enlarged	0 / mod enlarged / severely enlarged	0 / mod enlarged / severely enlarged
**Pneumonia**<br>Consolidation, type	0/acute/subacute/chronic	0/acute/subacute/chronic	0/acute/subacute/chronic	0/acute/subacute/chronic	0 /acute /subacute/chronic
Consolidation, distribution<br>Right side	0 /cran-lobe/caud-lobe<br><br>Focal/multifo/widespread	0 /cran-lobe/caud-lobe<br><br>Focal/multifo/widespread	0 /cran-lobe/caud-lobe<br><br>Focal/multifo/widespread	0 /cran-lobe/caud-lobe<br><br>Focal/multifo/widespread	0 /cran-lobe/caud-lobe<br><br>Focal/multifo/widespread
Consolidation, distribution<br>Left side	0 /cran-lobe/caud-lobe<br><br>Focal/multifo/widespread	0 /cran-lobe/caud-lobe<br><br>Focal/multifo/widespread	0 /cran-lobe/caud-lobe<br><br>Focal/multifo/widespread	0 /cran-lobe/caud-lobe<br><br>Focal/multifo/widespread	0 /cran-lobe/caud-lobe<br><br>Focal/multifo/widespread
Bact sample, case<br>2 sites from lesions	R cran-lobe /caud-lobe<br>L cran-lobe/caud-lobe	R cran-lobe /caud-lobe<br>L cran-lobe /caud-lobe	R cran-lobe /caud-lobe<br>L cran-lobe /caud-lobe	R cran-lobe /caud-lobe<br>L cran-lobe/caud-lobe	R cran-lobe /caud-lobe<br>L cran-lobe/caud-lobe
Bact sample, control<br>2 sites	R caudal lobe<br>L cranial lobe	R caudal lobe<br>L cranial lobe	R caudal lobe<br>L cranial lobe	R caudal lobe<br>L cranial lobe	R caudal lobe<br>L cranial lobe

Hist lung, case<br>4 samples per animal (preferably from lesions):<br>R and L cran-lobe,<br>R and L caud-lobe<br>(Cran-lobes in same vial, caud-lobes in same vial)	R: cran-lobe / caud-lobe<br><br>L: cran-lobe / caud-lobe	R: cran-lobe / caud-lobe<br><br>L: cran-lobe / caud-lobe	R: cran-lobe /caud-lobe<br><br>L: cran-lobe/caud-lobe	R: cran-lobe / caud-lobe<br><br>L: cran-lobe / caud-lobe	R: cran-lobe / caud-lobe<br><br>L: cran-lobe / caud-lobe
Hist lung, control<br>4 samples per animal	R: cran-lobe / caud-lobe<br><br>L: cran-lobe / caud-lobe	R: cran-lobe / caud-lobe<br><br>L: cran-lobe / caud-lobe	R: cran-lobe / caud-lobe<br><br>L: cran-lobe / caud-lobe	R: cran-lobe / caud-lobe<br><br>L: cran-lobe / caud-lobe	R: cran-lobe / caud-lobe<br><br>L: cran-lobe / caud-lobe
Hist ln, case and control	Tracheobronchial ln	Tracheobronchial ln	Tracheobronchial ln	Tracheobronchial ln	Tracheobronchial ln
Hist trachea, case	Trachea	Trachea	Trachea	Trachea	Trachea
Freeze lung tissue for PCR<br>Case:<br>sample from cran-lobe and caud-lobe (from lesions if there are any, in the same tube)<br><br>Control: L cran-lobe and R caud-lobe (in the same tube) (PCV2)	L cran-lobe/ caud-lobe<br>R cran-lobe/ caud- lobe	L cran-lobe/ caud-lobe<br>R cran-lobe/ caud- lobe	L cran-lobe/ caud-lobe<br>R cran-lobe/ caud- lobe	L cran-lobe/ caud-lobe<br>R cran-lobe/ caud- lobe	L cran-lobe/ caud-lobe<br>R cran-lobe/ caud- lobe
Freeze ln for PCR, case and control (PCV2)	Tracheobronchial ln	Tracheobronchial ln	Tracheobronchial ln	Tracheobronchial ln	Tracheobronchial ln
Freeze lung tissue, case<br>5 x 5 cm from lesion	Lesion	Lesion	Lesion	Lesion	Lesion
Photo cut surface (preferably from lesion)	Cut surface lesion	Cut surface lesion	Cut surface lesion	Cut surface lesion	Cut surface lesion
Tentative diagnosis					

Abbreviations: pur= purulent; bact= bacteriology; Cran-lobe= Cranial – and accessory lobe; Caud-lobe= Caudal lobe; R=Right; L=Left; ln=lymph node; Mod= moderately; Multifo= multifocal; Hist= Histology

Lesion type: acute = hyperemia and increased texture. Subacute = mixture between acute and chronic changes. Chronic = fibrosis, abscessation.

Instructions: circle around the alternative that fits the observation. Tick boxes when a task is done.
