# Supplementary material for: A descriptive study of acute outbreaks of respiratory disease in Norwegian fattening pig herds
Source: Acta Vet Scand. 2020 Jun 24;62:35. doi: 10.1186/s13028-020-00529-z (PMC7312110; doi:10.1186/s13028-020-00529-z)
Supplement: Supplementary file 2 — Additional file 2. Histology protocol. A scheme for a standardized histologic evaluation of sections from pigs’ lungs, pleura and tracheobronchial lymph nodes, including a description of section preparation. The scheme was compiled by members of the project group Grisefine lunger to be used in the study of acute respiratory disease outbreaks. [file 13028_2020_529_MOESM2_ESM.pdf]

Histology protocol. *A scheme for a standardized histologic evaluation of sections from pigs' lungs, pleura and tracheobronchial lymph nodes. The scheme was compiled by members of the project group Grisefine lunger to be used in the study of acute respiratory disease outbreaks. Tissue samples from the lungs, pleura and lymph nodes are fixed and in 10% phosphate-buffered formalin and embedded in paraffin wax. Cut sections (2-3 µm) are stained with hematoxylin and eosin. Samples are, when possible, taken from bordering areas between normal and affected tissue with macroscopic lesions. Sections are examined by microscope (Leica 020-518.500 DM/LS).*

**Journal number**	**Lung no.1**			**Lung no. 2**			**Lung no. 3**			**Lung no. 4**			**Lung no. 5**		
**Case/control***	**Left Cran/caud***	**Right Cran/caud***	**Pleura**	**Left Cran/caud***	**Right Cran/caud***	**Pleura**	**Left Cran/caud***	**Right Cran/caud***	**Pleura**	**Left Cran/caud***	**Right Cran/caud***	**Pleura**	**Left Cran/caud***	**Right Cran/caud***	**Pleura**
**Neutrophils**															
**Fibrin**															
**Necrotic leukocytes**															
**Peribronchial/ peribronchiolar infiltration LC and PLC**															
**Interstitial bleeding**															
**Lymph node subcapsular granulocytes**															
**Other remarks**															
**Other slides****															

*Cross out the alternative that does not apply/was not evaluated

** If other sections have been evaluated, for instance sections of trachea, clarify sample type and findings.

Cran=cranial, caud= caudal, LC = lymphocytes, PLC = plasma cells

For every observation that fits the description in the form, tick the corresponding box.
