# Supplementary material for: A descriptive study of acute outbreaks of respiratory disease in Norwegian fattening pig herds
Source: Acta Vet Scand. 2020 Jun 24;62:35. doi: 10.1186/s13028-020-00529-z (PMC7312110; doi:10.1186/s13028-020-00529-z)
Supplement: Supplementary file 3 — Additional file 3. Details of sample handling and diagnostics. A document containing extended details of sample handling and laboratory diagnostic methods performed in the study of acute respiratory disease outbreaks. [file 13028_2020_529_MOESM3_ESM.pdf]

**Additional file 3 - Details of sample handling and diagnostics**

Included in this document are extended details of diagnostic procedures that were conducted in *A descriptive study of acute outbreaks of respiratory disease in Norwegian fattening pig herds.* Some details are repeated in the manuscript to ensure proper context.

*Bacteriology*

The samples were handled as part of the Norwegian Veterinary Institute (NVI) bacteriology department's routine diagnostic work.

Swabbed material from lungs and pleura was cultured on 5% sheep's blood on agar base including a cross-streak of β-toxic *Staphylococcus aureus* to help the growth of Nicotinamide adenine dinucleotide (NAD) dependent bacteria like *A. pleuropneumoniae* and incubated in a humidity chamber in 5% CO2. The swabs were additionally cultivated on blood agar for anaerobic incubation and on a cysteine lactose electrolyte deficient (CLED) agar for aerobic incubation.

Colonies were isolated by secondary culturing. Colony identification was verified by Matrix assisted laser desorption ionization time-of-flight (MALDI-TOF) mass spectrometry (MALDI Biotyper®, Bruker Daltonics, Bremen, Germany).

*Serotyping by whole genome sequencing*

Pure cultures of *A. pleuropneumoniae* isolated from the lungs and pleura were sampled for whole genome sequencing at Statens Serum Institut (SSI), Copenhagen, Denmark.

Extracted bacterial DNA was quantified using the Qubit (Invitrogen, Waltham, MA, USA), followed by library preparation with the Nextera XT DNA Library Prep Kit (Illumina Inc., San Diego, CA, USA) using manufacturers protocol. The libraries were sequenced on the NextSeq 550 platform (Illumina Inc., San Diego, CA, USA) to obtain paired-end 151 bp reads. The serovar was determined based on the presence of the serovar specific cps operons by local BLAST using CLC Genomic Workbench 11.0.1.

*Virology*

The pooled oral fluid samples were run in real-time PCR according to NVIs molecular biology department's in-house procedures. Nucleic acids were extracted using a Nuclisense easyMAG 2, (Biomerieux, Marcy-l'Étoile, France). PCRs were run in a Stratagene Mx3005P (Agilent Technologies, Santa Clara, California, USA), with respective positive and negative controls on each plate. A cycle threshold (Ct) value for SIV below 37 was considered positive. PCV2 qPCR is a quantitative test where results are given as measures nucleotide copies in 200 µL sample, calculated from repeated measures at different Ct values.

*Serology*

Details regarding the commercial tests that were used to analyze for agent specific antibodies:

| **Agent name** | **Kit name** | **Manufacturer** | **Sensitivity*** | **Specificity*** |
|---|---|---|---|---|
| *A. pleuropneumoniae* | ID Screen® APP Screening Indirect | IDvet, Montpellier, France | 82.9% | 99.6%. |
| Influenza A virus | ID Screen® Influenza A Antibody Competition Multi-species | IDvet, Montpellier, France | 69% at the recommended cutoff of 0.5 | 89% at the recommended cutoff of 0.5 |
| PRRSV | IDEXX PRRS X3 | IDEXX, Maine, USA | 98.8% | 99.9% |
| PRCV | SVANOVIR® TGEV/PRCV-Ab | Boehringer Ingelheim Svanova, Uppsala, Sweden | 93% | 97% |
| *M. hyopneumoniae* | *Mycoplasma hyopneumoniae* ELISA | Oxoid™, Cheshire, England | 100% | 98% |

PRRSV = Porcine Reproductive and Respiratory Syndrome Virus, PRCV = Porcine Respiratory Coronavirus

*The sensitivity and specificity of the serologic tests have not been evaluated on the Norwegian pig population and might deviate somewhat from the references used by the manufacturers.

The analyses were performed as described by the manufacturers. Plate reading was performed with a Thermo Multiscan EX.
